# Surgical approach and management outcomes for junction tuberculous spondylitis: a retrospective study of 77 patients

**DOI:** 10.1186/s13018-018-1021-9

**Published:** 2018-12-06

**Authors:** Huipeng Yin, Kun Wang, Yong Gao, Yukun Zhang, Wei Liu, Yu Song, Shuai Li, Shuhua Yang, Zengwu Shao, Cao Yang

**Affiliations:** 1 Department of Orthopedics, Union Hospital, Tongji Medical College, Huazhong University of Science and Technology, Wuhan, 430022 China; 2 Department of Orthopedics, First Hospital of Wuhan, Zhongshan Road, No.215, Wuhan, 430022 China

**Keywords:** Junction tuberculous spondylitis, Surgical management, Anterior debridement, Fusion, Kyphosis

## Abstract

**Background:**

Junction tuberculous spondylitis involves the stress transition zone of the spine and has a high risk of progression to kyphosis or paraplegia. Problems still exist with treatment for spinal junction tuberculosis. This study investigated the surgical approach and clinical outcomes of junction spinal tuberculosis.

**Methods:**

From June 1998 to July 2014, 77 patients with tuberculous spondylitis were enrolled. All patients received 2–3 weeks of anti-tuberculous treatment preoperatively; treatment was prolonged for 2–3 months when active pulmonary tuberculosis was present. The patients underwent anterior debridement and were followed up for an average of 29.4 months clinically and radiologically.

**Results:**

The cervicothoracic junction spine (C7-T3) was involved in 15 patients. The thoracolumbar junction spine (T11-L2) was involved in 39 patients. The lumbosacral junction spine (L4-S1) was involved in 23 patients. Two patients with recurrence underwent reoperation; the drugs were adjusted, and all patients achieved bone fusion. The preoperative cervicothoracic and thoracolumbar kyphosis angle and lumbosacral angle were 31.4 ± 10.9°, 32.9 ± 9.2°, and 19.3 ± 3.7°, respectively, and the corresponding postoperative angles were ameliorated significantly to 9.1 ± 3.2°, 8.5 ± 2.9°, and 30.3 ± 2.8°. The preoperative ESR and C-reactive protein level of all patients were 48.1 ± 11.3 mm/h and 65.5 ± 16.2 mg/L which decreased to 12.3 ± 4.3 mm/h and 8.6 ± 3.7 mg/L at the final follow-up, respectively. All patients that had neurological symptoms achieved function status improvement at different degrees.

**Conclusion:**

For spinal tuberculosis of spinal junctions, anterior debridement, internal fixation, and fusion can be preferred and achieved. If multiple segment lesions are too long or difficult for operation of anterior internal fixation, combining posterior pedicle screw fixation is appropriate.

## Background

Over the past decades, the incidence of spinal tuberculosis has continued to increase due to population growth, acceleration of mobility, and HIV infection and spread [1]. Spinal tuberculosis can result in serious consequences without proper therapy in time. Although treatment with powerful anti-tuberculosis drugs has been used, surgical management is also critical. Advanced concepts of surgical procedures have evolved over the years, but there is still debate [2].

Surgical strategies of spinal tuberculosis are varied and include single or staged, anterior or posterior, and anterior-posterior or posterior-anterior combined operations [3]. Determining the optimal operative method is crucial, especially for junction spinal tuberculosis. The anterior segment, the weight-bearing area of the vertebral column, is preferred for spinal tuberculosis infection. Destruction of the anterior column alters the biomechanics and stability of the spine, which increases the risk of kyphosis and paraplegia progression in patients with junction spinal tuberculosis [4, 5]. The anterior approach has gradually become the main operative choice for spinal tuberculosis because it can directly reach the lesion site with a larger operative horizon to completely remove lesions, accomplishing the most important part of tuberculosis therapy. Moreover, full neural decompression, ample spinal stability reconstruction, and enough deformity correction can be achieved in one stage [6]. Although the posterior approach, which is used routinely in spinal operation, may show some advantages, it damages the residual normal structure against spinal stability and disease healing [7]. Junction tuberculous spondylitis involves the stress transition zone of the spine resulting in a high risk of progression to kyphosis or paraplegia. Thus, anterior rather than posterior approach seems to be preferred.

Therefore, we investigated clinical outcomes of the anterior procedure for treating patients with junction spinal tuberculosis, including cervicothoracic, thoracolumbar, and lumbosacral junctions.

## Methods

### Patient

From June 1998 to July 2014, 77 patients (age range18–72 years, with an average age of 35.2 ± 18.2 years) with junction tuberculous spondylitis, including 38 men and 39 women, who underwent anterior debridement, strut grafting, and instrumentation in our hospital enrolled the study. Spinal tuberculosis was diagnosed based on patients’ symptoms (local pain and percussion pain accompanied with fever, night sweats, and neurological dysfunction), laboratory results (T-spot, tuberculosis antibody, erythrocyte sedimentation rate [ESR], and C-reactive protein [CRP]) and radiologic findings (radiography, computed tomography, and magnetic resonance imaging) and was confirmed by postoperative pathology examinations. Imaging studies showed vertebral body destruction, intervertebral space collapse, kyphosis, paravertebral abscess, and intraspinal invasion. Regarding vertebral damage, 15 patients had cervicothoracic junction damage (C7-T3), 39 patients had thoracolumbar junction damage (T11-L2), and 23 patients had lumbosacral junction damage (L4-S1) (Table 1). Patients who had previously undergone surgery for TB or whose damage segments did not invade spine junction were excluded the study. The same surgeons reviewed the surgical indications and performed the procedures.

**Table 1:** Summary of the patients’ data

General data		Cervicothoracic	Thoracolumbar	Lumbosacral
Number of patients		15	39	23
Males/females		7/8	18/21	13/10
Mean age (years)		40.1 ± 10.3	45.2 ± 12.7	49.2 ± 14.0
Number of damaged segments	2	10	23	16
	≥ 3	5	16	7

Written informed consent was obtained from all patients, and the study protocol was approved by the Institutional Ethics Review Board of Tongji Medical College, Huazhong University of Science and Technology.

### Treatment method

#### Preoperative preparation

Routine chest radiography, sputum smear examination, and culture were performed preoperatively to screen active pulmonary tuberculosis. All patients received at least 2–3 weeks of first-line anti-tuberculous treatment (rifampicin 0.3 g, isoniazid 0.45 g, and ethambutol 0.75 g) preoperatively, which was prolonged to 2–3 months for the existence of active pulmonary tuberculosis. Moreover, supporting therapy and symptomatic treatment were conducted when necessary. Operations were not performed until the symptoms improved, and the ESR decreased to normal or close to normal.

#### Surgical approach

##### Cervicothoracic junction

**Fig. 1:**
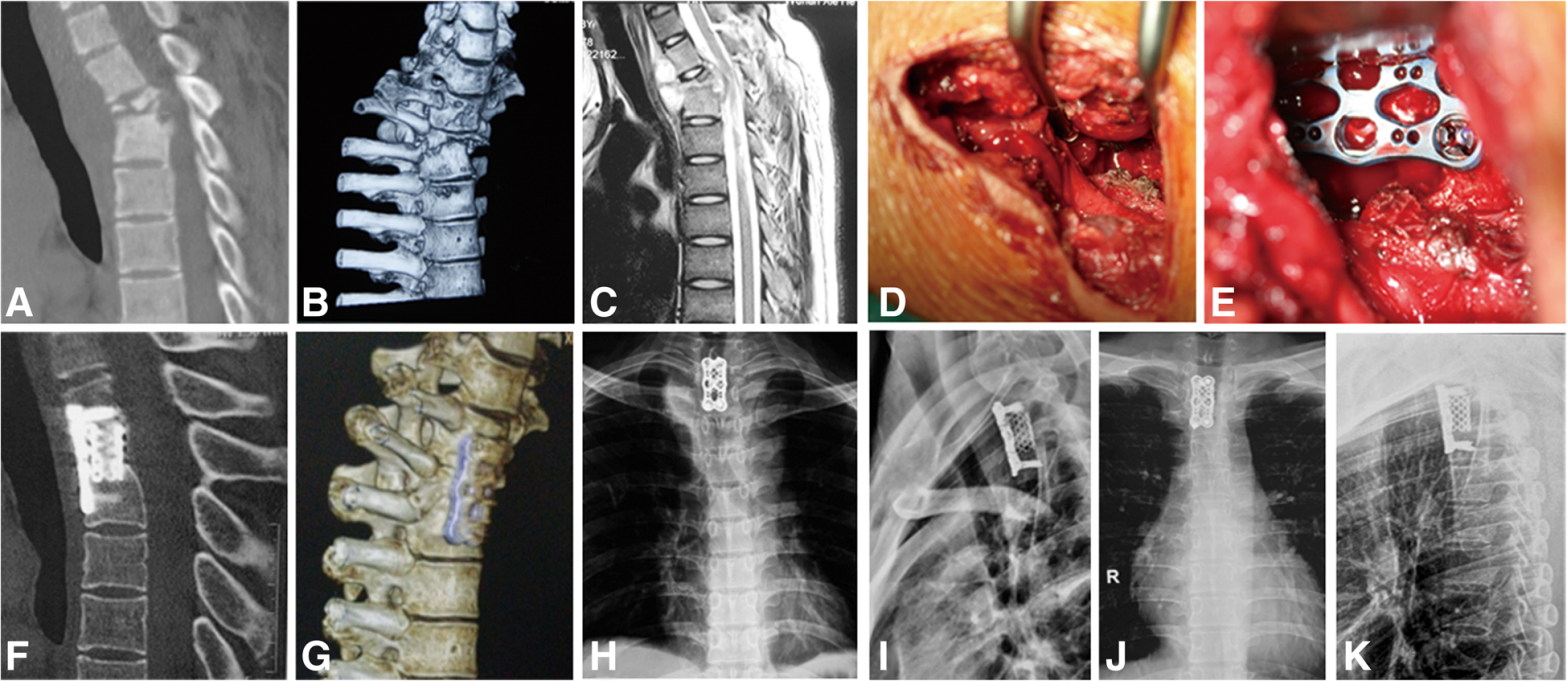
Nineteen-year-old boy with cervicothoracic tuberculosis (T1-3 level). **a**, **b** Preoperative computed tomography scan and three-dimensional reconstruction demonstrate vertebral body destruction with kyphosis. **c** Preoperative magnetic resonance imaging scan. **d**, **e** Intraoperative debridement, internal fixation, and fusion with titanium cage strut. **f**–**i** Radiographs postoperatively showing well-positioned internal fixation and improved kyphosis. **j**, **k** Radiographs showing satisfactory focal clearance and strut graft stability at the final follow-up

Patients were instructed to lay in supine position. A standard anterior approach was used, and an L-shaped incision was made. Skin and subcutaneous tissues were dissected layer by layer. The interclavicular ligament was cut at the upper edge of the sternum, while the sternoclavicular ligament was retained to avoid instability of the sternoclavicular joint. The sternal manubrium was fenestrated to expose the lesions based on the damaged segments, and the cutoff parts were reserved as autologous bone particles. After blunt dissection of the space between the sternal manubrium and mediastinum was performed, the prevertebral tissue was pushed to the side, and the lesion sites were reached through the gap. The abscess was aspirated by a thick needle for a culture specimen. The tuberculosis stove was completely removed, while normal vertebral bone tissue was retained. Interbody fusion and anterior internal fixation with a titanium plate and mesh cage were performed to recover the normal spinal curvature of patients with kyphosis (Fig. 1).

##### Thoracolumbar junction

**Fig. 2:**
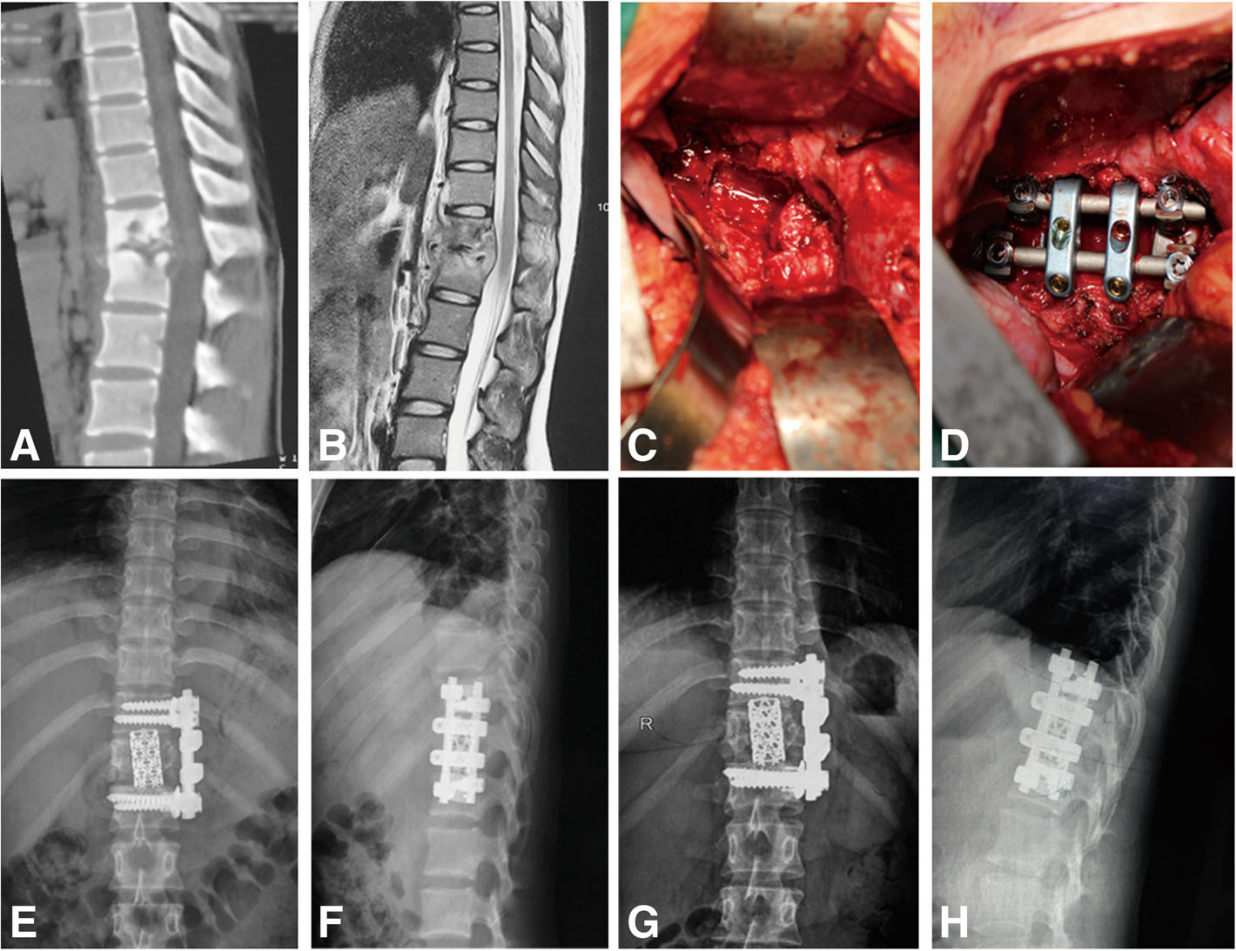
Twenty-year-old girl with tuberculosis (T12-L1 level). **a** Preoperative computed tomography scans demonstrate vertebral body destruction. **b** Preoperative magnetic resonance imaging scan shows vertebral body destruction and abscess. **c**, **d** Intraoperative debridement, titanium cage strut fusion, and internal fixation. **e**, **f** Postoperative radiographs show well-positioned internal fixation. **g**, **h** Radiographs showing satisfactory focal clearance and strut graft stability at the final follow-up

Patients were instructed to lay in lateral position so the severe lesions were readily accessible. Transthoracic patients received tracheal intubation with a double lumen tube, and the side lobe was collapsed intraoperatively. Skin and subcutaneous tissues were dissected layer by layer using only an incision of the oblique costal margin. Then the lesion was peeled from the pleural cavity using the retroperitoneal approach. The paravertebral abscess was aspirated with a thick needle as a culture specimen, followed by systematic completed debridement of the intervertebral space without normal vertebral bone tissue. An abscess in the opposite direction was drawn and flushed repeatedly through the vertebral body defect. Autogenous bone and graft fusion with a titanium cage strut combined with an anterior vertebral screw-rod internal fixation system were used to recover the normal spinal curvature of patients with kyphosis. A closed thoracic drainage tube was placed for thoracotomy. The diaphragm was sutured when necessary (Fig. 2).

##### Lumbosacral junction

**Fig. 3:**
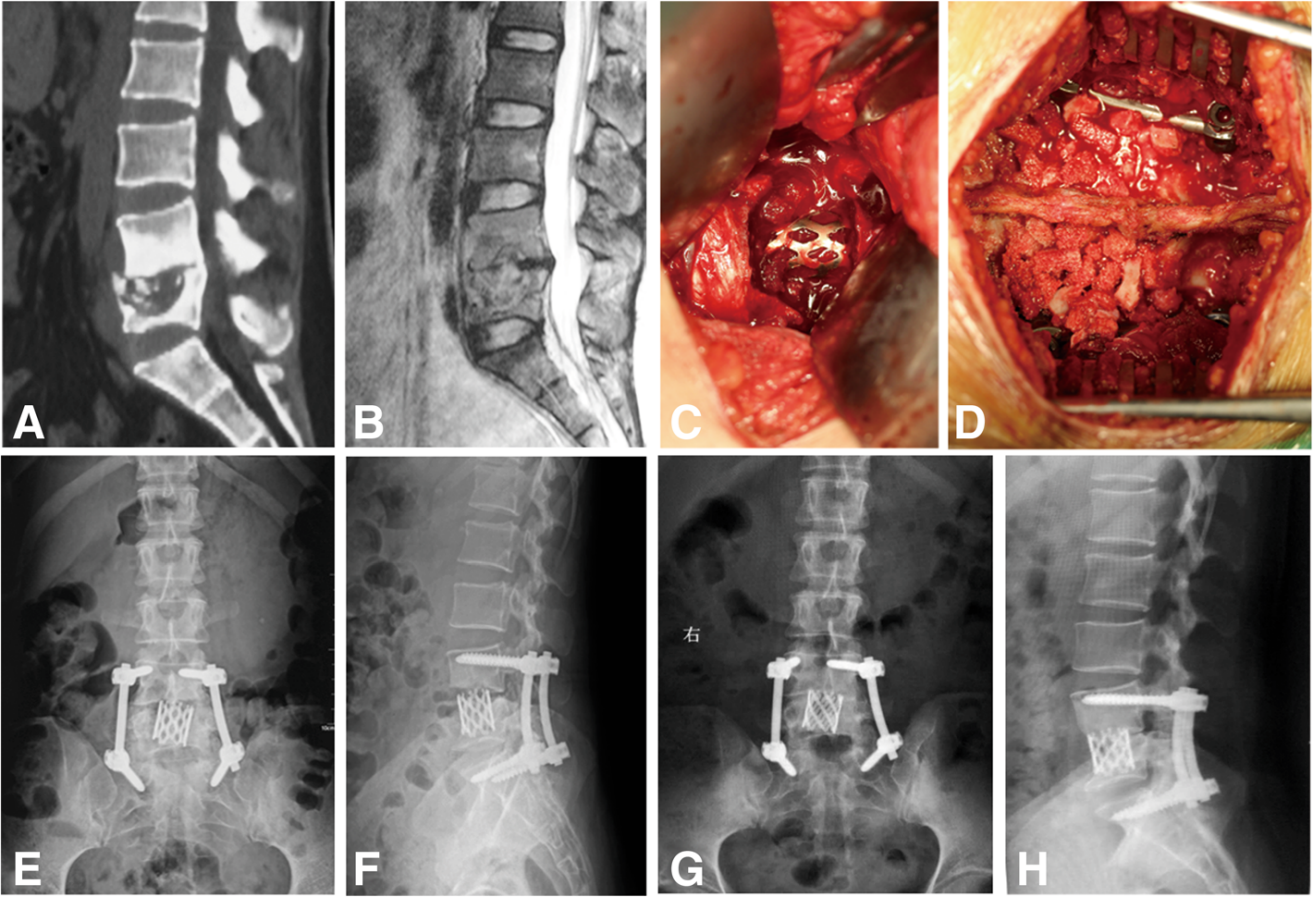
Twenty-seven-year-old woman with lumbosacral tuberculosis (L4-S1 level). **a**, **b** Preoperative computed tomography and magnetic resonance imaging scans demonstrate vertebral body destruction and abscess. **c** Anterior debridement and titanium cage strut fusion. **d** Posterior internal fixation and graft fusion. **e**, **f** Postoperative radiographs show well-positioned internal fixation. **g**, **h** Radiographs show satisfactory focal clearance and strut graft stability at the final follow-up

Patients were instructed to lay in supine position. Skin and subcutaneous tissue were cut along the abdominal transverse line. Internal organs were moved to the side to expose the lesion in the retroperitoneal space. The abscess and tuberculotic lesions were removed thoroughly, while normal vertebral bone tissue was retained. Different spinal positions and equipment combined with autogenous iliac or allograft bone fusion, supplemented by titanium cage support, were used to recover the normal spinal curvature of patients with kyphosis. If S1 was severely damaged or complex vascular structure affects anterior fixation, patients then need to be placed in the prone position for pedicle screw-rod internal fixation via a posterior midline approach (Fig. 3).

For all three junction areas, patients underwent thorough debridement to remove the pus liquid, abscesses, tuberculous granulomas, caseous necrotic substances, and necrotic bones after the lesions were found. The destroyed intervertebral disc and vertebral body were also removed. The bone bridge, sclerotic bone, and reactive bone were curetted gently, and this was repeated until fresh bone bled, and no lacuna was left. The wound was washed repeatedly with a dilute povidone-iodine solution and saline, and 3–4 g of streptomycin powder was administered. The drainage tube was placed postoperatively, and culture specimens were sent for pathological examination.

#### Postoperative treatment

Conventional electrocardiographic monitoring and anti-infection and anti-tuberculosis treatment were provided. A drainage tube was placed for 2 days and removed until the 24-h drainage flow was < 50 mL. The closed thoracic drainage tube was clamped until the drainage flow was < 100 mL for three consecutive days and removed until complete lung expansion was confirmed by radiography. The drainage time was extended in patients with penetrable pus cavities. Nutritional support was provided in patients with postoperative anemia, low serum albumin levels, or loss of appetite. Patients were encouraged to perform ambulation in the early postoperative period. After hospital discharge, anti-tuberculosis therapy was maintained for 9–12 months.

### Evaluation of clinical outcomes

All patients were followed up at 3, 6, 9, and 12 months postoperatively and then once annually. Outcomes included clinical manifestations, complications, and recurrence. Radiographs of the spine in anteroposterior and lateral positions were obtained postoperatively and during outpatient follow-up to determine the condition of bone fusion, kyphosis angle and lumbosacral angle, and physiological curve and to assess for displacement, loosening, or fracture of the bone with internal fixation and grafting. The ESR and CRP were measured to evaluate the activity of tuberculosis. Patients underwent a blood test locally 15 days later to monitor hepatic and renal function.

### Statistical analysis

Data were analyzed using the independent sample *t* test and SPSS statistical software (IBM Corp.). *p* values < 0.05 were considered statistically significant.

## Results

### Clinical results

All patients underwent operation successfully. Patients were followed up for 12–36 months (average, 29.4 ± 8.2 months). The symptoms of infection were alleviated and disappeared at 3–6 months postoperatively in all patients. All patients recovered activities of daily living at the final follow-up.

Among 77 patients, two experienced recurrence, and bone fusion was achieved after the second anterior radical debridement and the adjustment of anti-tuberculosis drugs. Unilateral sympathetic nerve injuries manifested as an increased skin temperature and less sweating in six thoracolumbar cases. In half of these patients, symptoms disappeared 2 or 3 days postoperatively. Two patients recovered within 3 months follow-up; the remaining patient had irreversible neurological damage. The screw used for internal fixation cut into the intervertebral space in two patients. As bone fusion was finally achieved and no obvious clinical symptoms developed, no additional treatment was performed. The other patients all achieved bone fusion.

### Laboratory data

The preoperative ESR and CRP level of all patients were 48.1 ± 11.3 mm/h and 65.5 ± 16.2 mg/L, which decreased to 12.3 ± 4.3 mm/h and 8.6 ± 3.7 mg/L at the final follow-up, respectively (Table 3).

### Neurologic function

Forty-eight patients had neurological symptoms manifesting as lower limb weakness, girdle sensation or associated numbness, and paresthesia. Neurological function was evaluated by the Frankel classification and is listed in Table 2. All patients achieved function status improvement at different degrees.

**Table 2:** Preoperative and postoperative neurological status by the Frankel score system (*n* = 48)

	Cervicothoracic (*n* = 9)			Thoracolumbar (*n* = 28)			Lumbosacral (*n* = 11)		
	PRE	POST	FFU	PRE	POST	FFU	PRE	POST	FFU
A				2					
B	2	1		3	2				
C	2	2		10	6	1	3		
D	4	3	3	9	12	7	4	4	2
E	1	3	6	4	8	20	4	7	9

*PRE* preoperative, *POST* postoperative, *FFU* final follow-up

### Radiological data

The preoperative cervicothoracic and thoracolumbar kyphosis angle and lumbosacral angle were 31.4 ± 10.9°, 32.9 ± 9.2°, and 19.3 ± 3.7° respectively. The corresponding postoperative angles were ameliorated significantly to 9.1 ± 3.2°, 8.5 ± 2.9°, and 30.3 ± 2.8°. At the final follow-up, only a small loss of correction was observed, as shown in Table 3.

**Table 3:** Laboratory data of all patients

	ESR (mm/h)		CRP(mg/L)		Kyphosis angle (lumbosacral angle in lumbosacral) (°)		
	PRE	FFU	PRE	FFU	PRE	POST	FFU
Cervicothoracic	48.7 ± 11.8	14.1 ± 3.6	62.3 ± 16.6	7.5 ± 3.9	31.4 ± 10.9	9.1 ± 3.2	10.2 ± 3.2
Thoracolumbar	47.5 ± 10.7	11.5 ± 4.2	66.6 ± 15.6	9.0 ± 3.6	32.9 ± 9.2	8.5 ± 2.9	9.9 ± 3.1
Lumbosacral	48.9 ± 12.5	12.2 ± 4.6	65.8 ± 17.4	8.4 ± 3.8	19.3 ± 3.7	30.3 ± 2.8	28.3 ± 2.3
Total	48.1 ± 11.3	12.3 ± 4.3	65.5 ± 16.2	8.6 ± 3.7			

*PRE* preoperative, *FFU* final follow-up, *POST* postoperative, *ESR* erythrocyte sedimentation rate, *CRP* C-reactive protein

## Discussion

The anterior approach enables surgeons to reach the lesion site directly, and a single incision can be used to perform multiple operations. Operators also have a more spacious and a direct field of vision, simplifying the operative procedures [8]. Moreover, it is a convenient way to completely remove tuberculotic substances from the lesion vertebra and paravertebral abscesses, to safely decompress soft oppression of the spinal cord, and to fully perform spinal canal decompression. As a result, a guaranteed procedure and limited tissue trauma are benefits of the anterior approach. Additionally, furthest retention of the posterior column structure maintains spinal stability. Anterior debridement combined with strut grafting can provide a suitable host bed to simulate vertebral bone fusion, and instrumentation by titanium plate and mesh stabilizes biomechanical properties of the spine, especially at the cervicothoracic, thoracolumbar, and lumbosacral junctions where stress is concentrated. This will reduce the risk of postoperative kyphosis and improve the surgical cure rate of junction spinal tuberculosis.

Several important structures are located in front of the cervicothoracic junction. Deep exposure of the affected vertebral body via the anterior approach may damage these structures, leading to serious complications. Therefore, specialized anatomical knowledge and expert surgical skills are required, which creates more of a challenge for surgeons. Zeng et al. compared the clinical curative effect of cervicothoracic spinal tuberculosis by anterior, anterior-posterior, and posterior approaches, and they found that the postoperative local recurrent deformity rate for the simple anterior approach was highest [5]. However, other studies have confirmed the curative effect of the anterior approach in cervicothoracic disease, especially in spinal tuberculosis [9–11]. The posterior approach to the cervicothoracic junction is disadvantageous because of the destabilization effect, inadequate visualization of the pathology, and need for a long posterior construct to restore stability. In our study, all patients with cervicothoracic spinal tuberculosis underwent anterior debridement, strut grafting, and instrumentation except one patient with four damaged segments (C7-T3) received posterior pedicle screw fixation. The sternum was partially resected according to the level of the thoracic position needed to reach the surgical site. All patients recovered well at the final follow-up without breakage or transposition of the implant or kyphosis recurrence.

The thoracolumbar junction is the most concentrated part of longitudinal load stress, as the vertebral body and retinaculum bear most of the body weight, and it is a predilection site of spinal tuberculosis. Thus, paravertebral abscesses often develop here. Anterior debridement and strut grafting enable surgeons to treat thoracolumbar spinal tuberculosis directly and thoroughly, which is more favorable for biomechanical reconstruction. Zhao et al. described patients in whom the thoracolumbar junction was affected and showed that the simple anterior operation corrected kyphosis and remained corrected until the last follow-up [12]. Cavuşoğlu et al. reported that patients with thoracolumbar spinal tuberculosis treated by anterior debridement and autogenous fibular grafting had good clinical and radiographic curative effects [13]. The anterior approach to thoracolumbar spinal tuberculosis was further modified to reduce operative injury [14]. However, posterior instrumentation may play an important role in multilevel thoracolumbar tuberculosis. Qureshi et al. performed radical debridement, strut grafting, and anterior instrumentation with pedicle screw fixation in patients with multilevel thoracolumbar tuberculosis. They found that recurrent deformity occurred only in simple anterior operation [15]. These results suggest that posterior instrumentation is required to enhance spinal stability and maintain correction of kyphosis when tuberculosis invades multiple segments. Compared with the posterior approach, the anterior approach shows efficacious and thorough debridement under direct vision, ensuring the safety of the operation and confirming the clinical outcome [16–18]. Seven patients with multilevel thoracolumbar tuberculosis (two for four damaged segments and five for three damaged segments) in our study underwent the anterior approach combined with pedicle screw fixation, and others underwent only the anterior approach; all of them had a sturdy implant and favorable curative state.

Lumbosacral segments are constituted by the activity of the lumbar spine and no activity of the sacral spine, and the torso weight is concentrated in this segment; the sacroiliac joint is essential for effective load transfer between the spine and lower extremities. It is not difficult to perform anterior debridement and strut grafting via the retroperitoneal approach for lumbosacral spinal tuberculosis. However, anterolateral great vessels and occlusion of the iliac ala make it difficult to perform anterior instrumentation [19, 20]. Therefore, we used one-stage anterior debridement and strut grafting combined with posterior instrumentation. An anterior median abdominal incision was made into the rectus to access the extraperitoneal space without transection of the abdominal muscles; the wound was very small, and the lesion was visible. Pang et al.’s long-term follow-up study showed that patients with lumbosacral tuberculosis did not experience recurrence after single-stage posterior transforaminal lumbar debridement, interbody fusion, and posterior instrumentation, which may be because laminectomy and partial resection of the facet joints provide relative adequate surgical space, and local implantation of anti-tuberculosis drugs with saline irrigation intraoperatively eliminate tuberculosis [21]. However, Jin et al. confirmed in their retrospective studies that patients within exhaustive debridement had an inferior therapeutic effect and were associated with a high recurrence rate [22]. Jiang et al. retrospectively analyzed a group of patients with lumbosacral spinal tuberculosis and found that patients who underwent anterior debridement and posterior strut graft fusion with internal fixation achieved good clinical outcomes [23]. In our study, only two patients with three damaged lumbosacral segments received pedicle screw fixation and the other 21 patients underwent anterior debridement, strut grafting, and instrumentation. All patients had good bone fusion and a clinical curative effect at the final follow-up.

Additionally, strut grafting should be used as much as possible, especially in patients with a large defect. A titanium cage supplemented by autologous bone or allograft bone graft achieved satisfying outcomes in our study. Wang et al. found in a 5-year follow-up study that the titanium cage collapses to a certain degree, but it did not affect the operative effect [24]. Cavuşoğlu et al. also achieved a good operative effect using autogenous fibular grafting [13].

## Conclusions

In summary, we believe that the simple anterior approach for debridement, strut grafting, and instrumentation should be considered for cervicothoracic and thoracolumbar spinal tuberculosis. If the lesion segments are too long for anterior instrumentation, the posterior approach should be added to pedicle screw fixation. Anterior debridement and strut grafting combined with posterior internal fixation is needed at the lumbosacral junction because of difficulties with anterior fixation. Spinal tuberculosis is a systemic disease. Therefore, improvement of patients’ physical quality and immunity is required both before and after the operation. The operative method should be determined based on the specific circumstances of each patient and the operator’s proficiency level. Systemic anti-tuberculosis chemotherapy is essential to cure spinal tuberculosis. Lastly, comprehensive measures must be taken to improve the cure rate of spinal tuberculosis.

## Abbreviations

CRP: C-reactive protein
ESR: Erythrocyte sedimentation rate
FFU: Final follow-up
POST: Postoperative
PRE: Preoperative
